# Indents

**Published:** 1902-07-15

**Authors:** 


### INDENTS.

**☞ Brief Articles of Technical or Scientific Value will receive notice and comment. Contributions invited.**

To Preserve Hydrogen Dioxid—Add one dram of boric acid to four ounces of hydrogen dioxide to preserve it from decomposition.—*Dental Era.*

Tempering.—The *Engineer* states that steel tempered in a solution of carbolic acid in water, is much harder and springy than it would be if tempered in water alone.

Diagnosing Caries.—Separate the teeth to admit a thin polishing disk, with the disk remove the stained spot. If an opaque white spot still remains, caries has begun.—J. L. Williams, *Cosmos.*

Abscess Syringe.—Dr. Geo. T. Carpenter of Chicago recommends a flat thin point which will pass down along the root without injury to the gum in the treatment of pyorrhea alveolaris.—*Review.*

Haskels Babitt Metal.—

| DIE | | | COUNTER DIE. | | |
|---|---|---|---|---|---|
| ℞ | Copper parts | 1 | ℞ | Lead pts | 5 |
| | Antimony " | 2 | | Tin " | 1 |
| | Tin " | 8 | | | |

Nausea from a Rubber Plate.—Dr. L. P. Haskell suggests that a metal plate will often be worn without nausea by persons who will not wear a rubber plate, because it is cooler. tasteless and cleaner.—*Review*

Nòn-Irritant Oxyphosphate of Zinc.—Dilute the phosphoric acid with oil of cloves or water when mixing cement for pulp capping. This will harden and not irritate, if applied without pressure.—Dr. Starr in *Cosmos*.

Canal Treatment.—Disinfect canal and prepare for filling. Make a workable paste of powdered charcoal with creosote, and fill canal to apex, seal with gutta-percha. It has an anodyne and antiseptic action.—W. R. Hughes *Pacific Gazette*.

Gold Chloride.—Dr. W. V-B. Ames in the *Review*, recommends a solution of gold chloride as a cauterant for sensitive carious spots on roots of teeth, it is more penetrating and more durable, it produces a purplish black instead of a jet black.

Burns.—

| | | | |
|---|---|---|---|
| ℞ | Cocaine | grs. | xv |
| | Aristol | ℥ | j |
| | Olive Oil | ℥ | v |
| | Lanolin | ℥ | xv |

Mix and apply to burned surface.—*Cosmos*.

Devitilization of Pulp Remnants.—To a canal from which the greater part of the pulp has been removed apply a solution of chromic acid, and then neutralize with lime water. Before filling wipe out the canal with Eucalyptus or Cajeput Oil.—Dr. Williams in *International*.

Calcium Dioxid, Ca. $O_2$.—Which is made by dissolving Hydrogen per oxid in lime water, is said to have very efficient germicidal properties, and a tooth powder containing ten percent effectively sterilized carious teeth in thirty minutes of contact. S. Harnstien in —*Cosmos*.

CAVITY-CUTTER FOR ARTIFICIAL TEETH.—Dissolve gum camphor in turpentine, making a saturated solution. Shape cavity with carborundum wheel, then use engine and an old discarded engine-bur kept moistened with solution. You will find this will cut with great facility.—*J. A. Rockey, D.D.S.*

TO DEFINE CAVITY OF DECAY.—Place rubber dam on the tooth and apply full strength formaldehyde solution to the carious region, it will clearly define the extent of the softened tooth structure.—D. F. W. Low, in April *Cosmos*. Dr. J. J. Madden in the same issue says that he uses 25 % solution of Pyrozone, for the same purpose.

WHY AMALGAM FILLINGS CHANGE FORM.—Dr. J. B. Willmott says that it is caused by the uneven mixture of the amalgam mass or by unequal consolidation when packing it into the tooth. If the mercury is largely confined to the upper part of the filling it will in time pass into the lower part and produce an enlargement there and a corresponding shrinkage above.—April *Cosmos*.

MOUTH-WASH.—

Oil of cinnamon, gr. iv;
Oil of cloves, gr. iv;
Thymic acid, gr. iv;
Saccharin, gr. iv;
Oil of peppermint, gr. xxij;
Tincture of rhatany, gr. xxxvij;
Alcohol, 90°, ℥iij. Mix.

Sig.—One tablespoonful in a glass of water.

*Zahntechnische Reform.*

TO PREVENT REGULATING APPLIANCES FROM CUTTING THE MOUTH.—Wrap the wires with a long narrow strip of rubber, cut from a sheet of rubber dam, fasten the ends by holes punched in them, to convenient teeth or projections on the appliances. The ends of wires or screws may be covered with small tubing or pieces of rubber suitably perforated.—Mary E. Blake, in *Dental Review*.

Dry Tooth Brush.—Dr. Gallup, of Boston, recommends using a dry tooth brush with dry powder as it produces better cleansing of the teeth. It may by increased friction produce a more complete massaging of the gums, but we can hardly believe that as effective cleansing of the teeth can be accomplished as with the saponaceous fluids, which assist materially in dissolving mucus accretions on the proximal surfaces and depressions of the teeth.

Care of Hands.—In order to keep the hands smooth, Caspar recommends (in *Texas Medical Journal*) the following:

℞—Ol. rosæ, 15 drops;

Glycerini, 1 drachm;

Sp. myrciæ, 3 drachms;

Ol. cajuputi, 20 drops. Misce.

Sig.—To be used on the hands every night before retiring, and before going out in the cold air in the morning.—*Medical Fortnightly*.

Arsenical Antidote.—In cavities which it is impracticable to hermetically seal arsenical paste. After the paste has been sealed in a proximal cavity with a gutta-percha filling, place a pledget of cotton saturated with Weld's Chloride of Iron, in the interdental space and in contact with the gum at the cervical margin of the filling. Any arsenic which may leak out will be quickly converted by the iron solution into inert arsenite of iron.—*Office and Laboratory*.

To Check Hemorrhage.—Calcium chlorid, in dose of 8 to 16 grains every two to four hours, should be tried in all forms of persistent hemorrhage, especially hemoptysis, hematuria, and intestinal hemorrhage of typhoid fever,—for this salt increases the coagulability of the blood. It should be remembered, however, that this drug should not be used more than three days continuously, for its prolonged use decreases the coagulability of the blood.—*Medical Brief*.

An Amalgam Inlay.—The tooth was too frail for a gold filling, and to prevent the disintegration of the enamel margins by the free mercury I took an impression of the cavity and secured a plaster cart of the tooth and cavity, this was boiled in stearin to harden the margins so they would bear manipulation. The cavity was filled with amalgam and after hardening over night it was set in the tooth with cement in the usual way. Amalgam was used instead of porcelain because it was not so brittle, it fitted perfectly and the restoration of contour was good.—S. G. Perry in *Cosmos*.

Coffee as a Cause of Pyorrhea Alveolaris.—Dr. J. B. Ernsmere in the April *Cosmos* observes that the continuous use of coffee will eventually cause pyorrhea alveolaris ; and that all efforts to cure the disease as long as the habit is kept up will be futile, supposing that pyorrhea is due to the accumulation or rather non-illimination of waste products from the body, he cites the fact that coffee is usually forbidden patients suffering from gouty and rheumatic disease and should likewise be forbidden dental subjects. He cites several cases which have come under his supervision, which yielded readily to treatment when the coffee habit had been broken up.

POURING ZINC.—Dr. Chupein in *Dental Office and Laboratory*, says to test molten zinc with a dry white pine stick. If the hot metal chars or ignites the wood, the metal is too hot to pour.

A better test is to melt the zinc then let it cool until it begins to granulate, stirring all the time so as to have it uniformly hot, but just as soon as it begins to get mushy place it back on the fire and as soon at it becomes fluid, pour at once, pouring only enough in the mould to fill or cover the arch, let this stand for a few seconds or until a crust forms around the outside, then gradually add sufficient zinc to complete the die. This will prevent the gas from perforating or displacing the palatal portion of the die.—*Ed. Register*.

CAPSULE IMPLANTATION.—Select a trephine two-thirds the size of a normal root, and drill the process to the proper depth, about two-thirds as deep as the normal socket. Make a thin, seamless gold or silver capsule or thimble and fill with unvulcanized rubber. Then place it in the alveolar socket which has been slightly enlarged at the bottom. By making pressure on the rubber the walls of the capsule will be made to conform to the shape of the socket and become mechanically fixed. Remove the vulcanite and fit into the capsule a suitable continuous gum tooth with cement. The result is a firmly implanted non-absorbable root, and if antiseptic precautions have been taken, no suppuration will follow. By using cocain the operation can be made painlessly.—R. E. Payne, in *International Journal.*

To Make Inlays from Impressions That Will Fit the Cavities.—Varnish the impression of the inlay cavity with three coats of shellac varnish, before pouring. This will sufficiently enlarge the cavity model that it will make allowance for the platinum matrix, providing it does not exceed one thousandth of an inch in thickness. To get the impression of the cavity, prepare it for the inlay without undercuts; fill with modeling compound and when hard dress with strips as though it were a gold filling, then remove by inserting in the hard modeling compound the point of a small excavator, being careful not to injure the borders, varnish as above and pour with plaster compound, which will give a duplicate of the tooth cavity into which the platinum matrix can be burnished for a porcelain or gold inlay.—Dr. S. G. Perry in *Cosmos.*

A Gold Plating Outfit.—Provide a covered pint jelly glass, a battery dry cell, two pieces of insolated copper wire 22 guage, one-fourth ounce of Potassium Cyanide, and fifteen grains of gold chloride. Dissolve the Cyanide in two and one half ounces of warm distilled water, and add to it the gold chloride which has been previously dissolved in two and one half ounces of cold distilled water; place in the glass vessel, attach the copper wires to the positive and negative poles of the dry cell, remove two or three inches of the insulation, and place them in the solution. To the positive electrode attach a piece of pure gold, and, to the negative electrode, such articles as are to be plated Articles to be plated, should be first cleansed by polishing and immersing in a heated solution of Caustic Potash, which must be thoroughly removed by washing in clear water.—*Dental Era.*

Bandless Incisor Crown.—Trim root to conform to gum margins, presenting on its end a double concave surface. Tap the canal to proper depth and ream to size and shape for a flattened post. Fit a platino-iridium post accurately. Cut a disk of No. 40 platinum plate slightly larger than end of root and partially adapt to form of the root, and force the post through this cap to its place in the canal. Remove both post and cap, and then reverse the cap, replace the post, and carefully burnish the cap to fit the end of root accurately. Then remove the post and cap together by grasping with suitable pliers the post and edges of the platinum cap which are forced through when puncturing it by the post. Solder the cap to post by 20% platinum solder. Then replace on root and trim the cap nearly to root circumference, then burnish carefully to fit and trim accurately to circumference. Cut off post to proper length for crown. Select a perforated tooth crown such as the old Bonwill, or Howe and grind to place. Remove crown and take impression in compound with post in place, remove and pour model. To expidite removal of post from plaster model make a loosely fitting tube of tin foil on the root end of post, and slip it over the post just before pouring the impression. An articulating model may be made if necessary. Adjust the crown to post and attach with porcelain and set with gutta-percha.—A. E. Matterson in *Review.*

ANOMALIES.—A young man called for me to make a crown for a right lower lateral which had been broken off a year or more previously by accident, the nerve was extracted at the time and the root canal treated somewhat, but not filled. At this time the root was overlapped by the gum, upon examination, I found the canal had been effectually sealed by secondary dentine, beneath which was a living pulp. The second case: a lady complained of a right lower second molar which showed no signs of decay but her grievance was such that I drilled the mesio-distal fissure and filled it with cement, but the trouble was not remedied and the pulp was destroyed which gave temporary relief then there was a soreness until it was discovered that the tooth was split along this fissure entirely through the crown, it was extracted. This was about one year ago. A few days ago the same lady called with the same complaint against the same tooth on the left side, upon touching the mesio-distal fissure with an excavator a sharp pain was produced. Although this tooth like the other was apparently perfectly sound I determined to extract it at once which was done. It was found to be split directly through the whole tooth on the line of the fissure, I know these teeth to have been subjected to no traumatic injury at any time. The patient is about 38 years of age, of a rheumatic diathesis. The condition is either the result of malnutrition or of a failure of the two halves of the teeth to form a perfect union at the time of their developement.—"Old Q. in *Dental World*.

